# Tempera Decorations in Hospitals

**Published:** 1912-07-20

**Authors:** 


*To the Editor of* The Hospital.

### Tempera Decorations in Hospitals.

*To the Editor of* The Hospital.

Sir,—I have just read with great interest your article in a recent issue of The Hospital advocating the use of tempera decorations in hospitals. As I have for some years devoted special attention to the various styles of decoration adopted in hospitals, and as the matter is of some general interest, you may, perhaps, allow me a few remarks in criticism of some of the suggestions contained in the article.

Neither tempera nor fresco decoration is new so far as hospitals are concerned. In the hospitals of Italy, Greece, and Russia fresco decorative mural ornamentation is to be found, but, so far as I have experienced, it has never commended itself either to the superintendents and medical staff or to the in-patients. The repression of high lights and strong contrasts, which is a feature in fresco work, may appear to suit it admirably for hospital purposes, but, as a matter of fact, in countries where the direct sunlight is much stronger than here in England, the general appearance of a ward with frescoes is not attractive. This, in my opinion, is solely due to the repressive, subdued character of the fresco work itself, which is much more suitable for a church or a public meeting-hall than for a hospital ward or day-room. In England there are, of course, various other objections to the use of fresco. The climate, for instance, does not facilitate the crystallisation process, and artists find it singularly difficult to get proper tones owing to the slowness with which the plaster dries. So far as I am aware there are comparatively few examples of fresco work in England, and none of them is old enough to be taken as a test of the durability and lasting qualities of this kind of wall decoration. The beautiful work of Miss Emily Ford, which I understand has been more in the nature of an experiment, is very successful, but is as yet very modern.

If fresco work is adopted for a hospital day-room the colours chosen must be brighter and more reflective than those usually employed. The expense and cost of upkeep of such decorations will, I feel sure, prevent them from becoming popular in this country. With regard to tempera the case is different. Mural decorations in tempera stand the effects of climate and change of temperature very well, as witness the fine condition in which the wall paintings in the Bayswater Chapel remain. The objection to this kind of decoration is that the surface is not covered by an impermeable enamel as in the case of frescoes. Tempera surfaces, in order to obtain the broadest effects, are open and singularly porous, and as is well known a porous wall plaster easily absorbs odours and very probably (although I admit that this has not been demonstrated) germs as well. By covering the tempera-painted surface with an impervious enamel this objection will, of course, be removed, but in that case it is difficult to see where the superiority of tempera over other styles of mural decoration comes in, since such a covering varnish will, of course, enhance surface reflections and detract from the soberness and calm that are the characteristics of tempera work. My own opinion is that it will be much better to stick to simple wall washes, chosen so as to harmonise, and thereby to get a colour scheme which cannot be said to be decorative in the true sense of the word, rather than to attempt to embellish the walls by artistic designs, whether floral or figure. Exceptions may be made in the case of the committee room, the entrance lobbies, and perhaps the corridors.

In a sick-room or ward it is highly undesirable that there should be on the walls or anywhere in the room anything that can attract the attention of the patients in such a manner as to evoke psychological or psychiatrical stimuli. I have recently been struck by the fact that green is a favourite ward colour, whereas browns and blues, which are much less stimulating, are comparatively rarely used. From the patient's point of view I think this is a mistake. To deal fully with the matter here would take up too much space, but I trust you will allow me to return to it on some future occasion. It is just in the attention paid to these little details, which to the ordinary architect or hospital superintendent may seem of relatively little importance, that the superiority of certain hospital types above their rivals is so conspicuous. I question very much whether in London, for instance, with the possible exception of one or two large and one small institution, there is a hospital ward which may truly be described as ideal in so far as its colour scheme and restfulness are concerned.—I remain, yours truly,

"Your Special Commissioner."

